# Transition to Weight-Based High-Flow Nasal Cannula Use Outside of the ICU for Bronchiolitis

**DOI:** 10.1001/jamanetworkopen.2024.2722

**Published:** 2024-03-18

**Authors:** Robert J. Willer, Patrick W. Brady, Amy N. Tyler, Jennifer D. Treasure, Eric R. Coon

**Affiliations:** 1 Department of Pediatrics, Division of Pediatric Hospital Medicine, University of Utah School of Medicine, Primary Children’s Hospital, Salt Lake City; 2 University of Cincinnati College of Medicine, Cincinnati Children’s Hospital, Cincinnati, Ohio; 3 The Ohio State University College of Medicine, Nationwide Children’s Hospital, Columbus

## Abstract

**Importance:**

Most children’s hospitals have adopted weight-based high-flow nasal cannula (HFNC) bronchiolitis protocols for use outside of the intensive care unit (ICU) setting. Whether these protocols are achieving their goal of reducing bronchiolitis-related ICU admissions remains unknown.

**Objective:**

To measure the association between hospital transition to weight-based non-ICU HFNC use and subsequent ICU admission.

**Design, Setting, and Participants:**

This multicenter retrospective cohort study was conducted with a controlled interrupted time series approach and involved 18 children’s hospitals that contribute data to the Pediatric Health Information Systems database. The cohort included patients aged 0 to 24 months who were hospitalized with a diagnosis of bronchiolitis between January 1, 2010, and December 31, 2021. Data were analyzed from July 2023 to January 2024.

**Exposure:**

Hospital-level transition from ICU-only to weight-based non-ICU protocol for HFNC use. Data for the ICU-only group were obtained from a previously published survey.

**Main Outcomes and Measures:**

Proportion of patients with bronchiolitis admitted to the ICU.

**Results:**

A total of 86 046 patients with bronchiolitis received care from 10 hospitals in the ICU-only group (n = 47 336; 27 850 males [58.8%]; mean [SD] age, 7.6 [6.2] years) and 8 hospitals in the weight-based protocol group (n = 38 710; 22 845 males [59.0%]; mean [SD] age, 7.7 [6.3] years). Mean age and sex were similar for patients between the 2 groups. Hospitals in the ICU-only group vs the weight-based protocol group had higher proportions of Black (26.2% vs 19.8%) and non-Hispanic (81.6% vs 63.8%) patients and patients with governmental insurance (68.1% vs 65.9%). Hospital transition to a weight-based HFNC protocol was associated with a 6.1% (95% CI, 8.7%-3.4%) decrease per year in ICU admission and a 1.5% (95% CI, 2.8%-0.1%) reduction per year in noninvasive positive pressure ventilation use compared with the ICU-only group. No differences in mean length of stay or the proportion of patients who received invasive mechanical ventilation were found between groups.

**Conclusions and Relevance:**

Results of this cohort study of hospitalized patients with bronchiolitis suggest that transition from ICU-only to weight-based non-ICU HFNC protocols is associated with reduced ICU admission rates.

## Key Points

**Question:**

Are weight-based non–intensive care unit (ICU) high-flow nasal cannula (HFNC) protocols associated with reduced ICU admission?

**Findings:**

In this cohort study of 18 children’s hospitals with over 86 000 patients hospitalized with bronchiolitis, hospital-level adoption of a weight-based HFNC protocol was associated with a 6.1% reduction per year in ICU admission compared with ICU-only HFNC use.

**Meaning:**

Findings of this study showed an association between weight-based non-ICU HFNC use and decreased ICU admission among patients hospitalized with bronchiolitis.

## Teaser

This cohort study assesses changes in intensive care unit (ICU) admission rate, hospital length of stay, and use of noninvasive and invasive ventilation after the adoption of weight-based high-flow nasal cannula protocol in hospitalized patients aged 0 to 24 months.

## Introduction

High-flow nasal cannula (HFNC) is a common respiratory support modality in children hospitalized with bronchiolitis^1,2,3,4,5^; more than 50% of children were exposed to HFNC in a recent multicenter study.^6^ Use of HFNC was initially restricted to the intensive care unit (ICU), but over time it has expanded to pediatric wards to reduce ICU use for patients with bronchiolitis.^2,5^ Early protocols for ward-based HFNC applied flow rates according to age (eg, 8 L of flow for children younger than 12 months).^1^ An emerging alternative to age-based HFNC protocols are weight-based HFNC protocols (eg, 2 L of flow per kilogram of body weight).

The present study focused on weight-based non-ICU HFNC use for 2 principal reasons. First, multicenter studies have examined the transition to age-based non-ICU HFNC use, but similar studies for weight-based protocols are limited.^7,8^ Second, a recent survey of children’s hospitals that participate in the Pediatric Health Information Systems (PHIS) found that age-based HFNC protocols are being replaced by weight-based protocols; in a 2021 survey, 75% of hospitals with non-ICU HFNC protocols used weight-based flow rates^5^ compared with 27% of hospitals in a 2017 survey.^2^ The objective of this study was to measure the association between hospital transition to weight-based non-ICU HFNC use and subsequent ICU admission.

## Methods

We conducted a multicenter retrospective cohort study involving 18 children’s hospitals that contribute patient-level data to the PHIS database, including demographic characteristics, procedure codes, and discharge diagnosis codes. The University of Utah Institutional Review Board deemed this study exempt from review and the informed consent requirement because it was not human participant research. We followed the Strengthening the Reporting of Observational Studies in Epidemiology (STROBE) reporting guideline.

### Patient Characteristics

Patients aged 0 to 24 months who were hospitalized between January 1, 2010, and December 31, 2021, were included in the cohort if they had any *International Classification of Diseases, Ninth Revision (ICD-9)* or *International Statistical Classification of Diseases and Related Health Problems, Tenth Revision (ICD-10)* bronchiolitis code in any diagnostic position.^9,10^ Patients were excluded if they received care in the neonatal ICU or had a length of stay (LOS) greater than 30 days.

### Exposure and Outcomes

The study exposure was hospital-level transition from ICU-only to weight-based use of HFNC in non-ICU wards. Because HFNC use is not reliably documented in the PHIS database, we obtained data from our 2021 survey^5^ (which had an 82% response rate) to identify hospitals that restricted HFNC use to the ICU (ICU-only group) and hospitals that transitioned from using HFNC only in the ICU to adopting weight-based HFNC use in non-ICU wards (weight-based protocol group). Ten hospitals were included in the ICU-only group, and 8 hospitals were in the weight-based protocol group. Together, these 18 hospitals were the source of the patient-level data we obtained from the PHIS database. The PHIS database includes a hospital indicator, allowing linkage to the 2021 survey results and patient-level data.

The primary outcome was the proportion of patients with bronchiolitis admitted to the ICU. Secondary outcomes included mean total hospital LOS, the proportion of patients who received noninvasive positive pressure ventilation (NIPPV), and the proportion of patients who received invasive mechanical ventilation (IMV). Patients were classified as receiving NIPPV if they had an *ICD-9* or *ICD-10* procedure code for NIPPV and as receiving IMV if they had a procedure or supply code for mechanical ventilation and a pharmacy charge code for a neuromuscular blocking agent, in accordance with previously published definitions.^10,11,12^ A crosswalk available from the Centers for Medicare and Medicaid Services was used to convert previously published definitions that contained *ICD-9* codes to *ICD-10* codes.

### Changes in Outcomes 

We used a controlled interrupted time series approach to measure changes in outcomes associated with the transition from ICU-only to weight-based HFNC use, comparing 3 years before with 3 years after transition. The transition point for the weight-based protocol group was determined by survey responses.^5^ Because the ICU-only group did not have a true transition point, we established the transition point by matching the ICU-only hospitals with the weight-based protocol hospitals according to size (number of beds) and geographic location. Each ICU-only hospital was given the transition point of its matched weight-based protocol hospital. Next, we measured the immediate change at the transition point and the change in slope after the transition point.^13^

We identified the immediate change and the change over time associated with the transition by comparing changes in the slopes observed in the weight-based protocol group with changes observed in the ICU-only group.^14^ This approach reduces unmeasured confounding by controlling for other interventions and events concurrent with the study exposure.^14^

### Statistical Analysis

Statistical analyses were performed with 2 models: a mixed-effects linear regression model and a time series model. First, we ran the mixed-effects linear regression model using an interaction between time relative to transition and weight-based protocol adoption status, accounting for patient clustering within hospitals. We adjusted for patient age, sex, race, ethnicity, insurance type, and whether hospitalization occurred during the COVID-19 pandemic. We defined hospitalization during the COVID-19 pandemic as starting in April 2020 and ending in December 2021. Race and ethnicity (categorized as American Indian, Asian, Black, Hispanic and non-Hispanic, Native Hawaiian, White, and other [not specified]) were identified in hospitals and obtained from the PHIS database. Race and ethnicity data were collected and analyzed in the study because of prior research showing race-based disparities in receipt of bronchiolitis guideline–adherent care.^15^

Second, we used adjusted means for each outcome from the mixed-effects model as inputs for a controlled ordinary least squares time series model. We adjusted for autocorrelation with Newey-West SEs.^16^ Two-sided *P* < .05 indicated statistical significance. Analyses were performed from July 2023 to January 2024 using Stata, version 16 (StataCorp LLC).

## Results

A total of 86 046 patients with bronchiolitis received care at 10 hospitals in the ICU-only group (n = 47 336) and 8 hospitals in the weight-based protocol group (n = 38 710). Age and sex were similar between the ICU-only group (19 479 females [41.2%] and 27 850 males [58.8%]; mean [SD] age, 7.6 [6.2] years) and weight-based protocol group (15 865 females [41.0%] and 22 841 males [59.0%]; mean [SD] age, 7.7 [6.3] years) (Table 1). Hospitals in the ICU-only group vs weight-based protocol group had higher proportions of Black (26.2% vs 19.8%) and non-Hispanic (81.6% vs 63.8%) patients and patients with governmental insurance (68.1% vs 65.9%). Hospital characteristics including freestanding status, size, mean daily census, and geographic region were similar between the 2 groups (Table 2).

**Table 1.** Patient Demographic Characteristics by Group

Characteristic	HFNC use group, No. (%)		*P* value
	ICU only (n = 47 336)	Weight-based protocol (n = 38 710)	
Age, mean (SD), mo	7.6 (6.2)	7.7 (6.3)	.20
Sex			
Female	19 479 (41.2)	15 865 (41.0)	.58
Male	27 850 (58.8)	22 841 (59.0)	
Race^a^			
American Indian	737 (1.6)	149 (0.4)	<.001
Asian	866 (1.8)	643 (1.7)	
Black	12 414 (26.2)	7657 (19.8)	
Native Hawaiian	97 (0.2)	256 (0.7)	
White	26 012 (55.0)	21 181 (54.7)	
Other^b^	5059 (10.7)	4590 (11.9)	
Missing data	2151 (4.5)	4234 (10.9)	
Ethnicity^a^			
Hispanic	3672 (7.8)	8606 (22.2)	<.001
Non-Hispanic	38 630 (81.6)	24 707 (63.8)	
Missing data	5034 (10.6)	5397 (13.9)	
Insurance type			
Governmental	32 249 (68.1)	25 517 (65.9)	<.001
Nongovernmental	14 663 (31.0)	13 140 (33.9)	
Unknown	424 (0.9)	53 (0.1)	

Abbreviations: HFNC, high-flow nasal cannula; ICU, intensive care unit.

^a^ Race and ethnicity were identified in hospitals and obtained from the Pediatric Health Information Systems database.

^b^ Other category was not specified in the database.

**Table 2.** Hospital Characteristics by Group

Characteristic	HFNC use group, No. (%)		*P* value
	ICU only (n = 10)	Weight-based protocol (n = 8)	
Freestanding status	6 (60.0)	8 (100.0)	.09
Hospital size, No. of beds			.91
Mean (SD)	351 (69.6)	355 (141.7)	
<200	0	1 (12.5)	
200 to <300	2 (20.0)	2 (25.0)	
300 to <400	5 (50.0)	4 (50.0)	
400 to <500	3 (30.0)	0	
500 to <600	0	0	
600 to <700	0	1 (12.5)	
Daily census, mean (SD) No.	245 (17.6)	254 (109.0)	.85
Geographic region			
Midwest	4 (40.0)	2 (25.0)	.92
Northeast	2 (20.0)	1 (12.5)	
South	2 (20.0)	2 (25.0)	
West	2 (20.0)	3 (37.5)	

Abbreviations: HFNC, high-flow nasal cannula; ICU, intensive care unit.

For the ICU-only group, we observed a 1.8% (95% CI, 1.0%-2.6%) increase per year in ICU admission, a 2.5% (95% CI, 2.1%-2.9%) increase per year in NIPPV use, and an immediate increase in NIPPV use of 1.6% (95% CI, 0.8%-2.4%) (Table 3 and Figure). We observed no significant changes in mean total LOS or the proportion of patients receiving IMV. For the weight-based protocol group, we observed a 4.2% (95% CI, 6.8%-1.7%) reduction per year in ICU admission, a 0.1-day (95% CI, 0.0- to 0.1-day) increase in mean total LOS, and a 4.2% (95% CI, 2.6%-5.7%) immediate increase in NIPPV use. We observed no significant changes in the proportion of patients receiving IMV.

**Table 3.** Outcomes by Group

Outcome and HFNC use group	Immediate change, % (95% CI)	Change in slope, % (95% CI)
ICU only		
ICU admission	0.9 (−0.7 to 2.5)	1.8 (1.0 to 2.6)^a^
Mean (SD) total LOS, d	−0.0 (−0.2 to 0.1)	0.1 (−0.0 to 0.2)
NIPPV	1.6 (0.8 to 2.4)^a^	2.5 (2.1 to 2.9)^a^
IMV	−0.3 (−1.1 to 0.6)	−0.2 (−0.8 to 0.4)
Weight-based protocol		
ICU admission	1.6 (−1.5 to 4.7)	−4.2 (−6.8 to −1.7)^a^
Mean (SD) total LOS, d	0.0 (−0.1 to 0.1)	0.1 (0.0 to 0.1)^a^
NIPPV	4.2 (2.6 to 5.7)^a^	1.0 (−0.3 to 2.3)
IMV	−0.2 (−1.3 to 0.8)	−0.6 (−1.2 to 0.1)
Outcome associated with transition		
ICU admission	0.7 (−2.8 to 4.2)	−6.1 (−8.7 to −3.4)^a^
Mean (SD) total LOS, d	0.0 (−0.1 to 0.2)	−0.0 (−0.1 to 0.1)
NIPPV	2.5 (0.8 to 4.3)^a^	−1.5 (−2.8 to −0.1)^a^
IMV	0.1 (−1.3 to 1.4)	−0.4 (−1.3 to 0.5)

Abbreviations: HFNC, high-flow nasal cannula; ICU, intensive care unit; IMV, invasive mechanical ventilation; LOS, length of stay; NIPPV, noninvasive positive pressure ventilation.

^a^ Statistically significant results.

**Figure.**
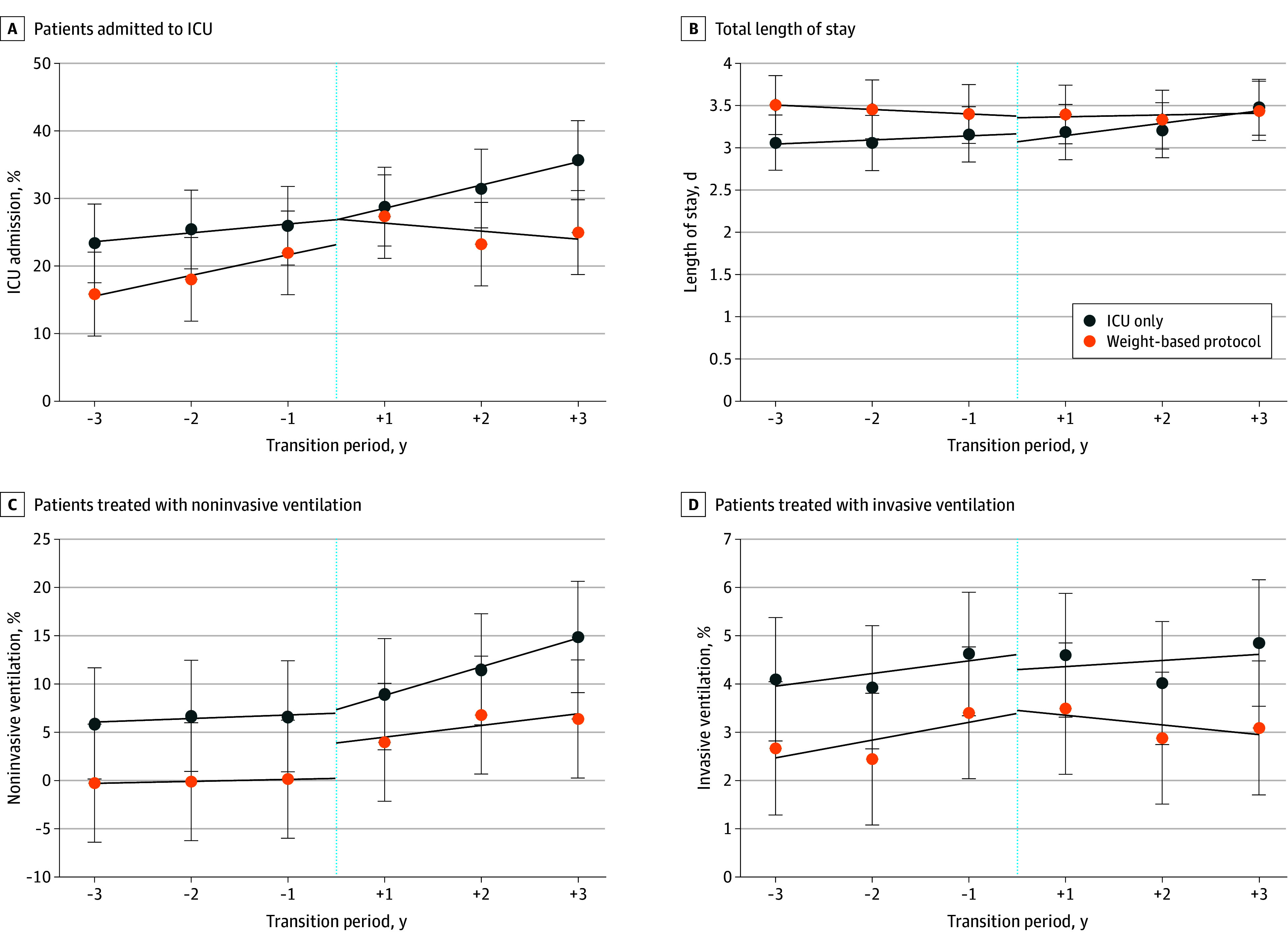
Comparison of Outcomes Between Intensive Care Unit (ICU)–Only and Weight-Based Protocol High-Flow Nasal Cannula (HFNC) Use The dashed line indicates the transition point from using HFNC at ICU only to adopting a weight-based HFNC protocol. Error bars represent 95% CIs.

We found that hospitals that transitioned to the weight-based protocol experienced a 6.1% (95% CI, 8.7%-3.4%) decrease per year in ICU admissions, a 1.5% (95% CI, 2.8%-0.1%) decrease per year in NIPPV use, and a 2.5% (95% CI, 0.8%-4.3%) immediate increase in NIPPV use compared with ICU-only hospitals (Table 3 and Figure). We found no significant changes in mean hospital LOS or IMV use associated with the transition.

## Discussion

We observed a significant year-over-year decrease in the proportion of patients admitted to the ICU associated with transition from an ICU-only to a weight-based non-ICU HFNC protocol. This finding is timely given the well-described pattern of increasing ICU admission rates and hospital costs for children with bronchiolitis.^9,10,17^ Randomized clinical trials that have examined the outcomes of weight-based protocols in children hospitalized with bronchiolitis were not powered to measure a difference in ICU admission.^18,19,20,21^ The present observational study starts to fill this gap in evidence, suggesting that weight-based protocols may be effective in reducing ICU use for children hospitalized with bronchiolitis.

It is important to consider alternative explanations for the association between a decrease in ICU admissions and weight-based HFNC protocols. For example, it is possible that changes over time in bronchiolitis viral etiologies or decreasing illness severity could also affect ICU admission rates for children hospitalized with bronchiolitis. For either of these factors to explain the findings, viral etiologies and/or illness severity would have to be fundamentally different at hospitals with ICU-only vs weight-based protocol HFNC use. A comparison by matching hospital size and geographic region mitigates this possibility.

Transition to weight-based protocols was not associated with changes in LOS or IMV. These findings augment an existing body of evidence that suggests use of HFNC is safe for use outside the ICU setting.^8,22^ While we found that NIPPV use was lower over time in the weight-based protocol group vs the ICU-only group, NIPPV use appeared to increase overall in both groups. This finding is consistent with previous literature that showed a 6- to 7-fold increase in NIPPV use in children’s hospitals since 2010 and a 15-fold increase in a nationally representative population since 2000.^9,10,17^ For hospitals that have transitioned to non-ICU use of HFNC, one could imagine a pathway in which intervention begets intervention. When a patient already receiving HFNC outside the ICU setting is transferred to the ICU, it is natural to escalate the child to NIPPV. Previously, when HFNC was limited to the ICU, this same patient might have received HFNC at the time of ICU transfer without further escalation to NIPPV. This clinical scenario could partially explain the observed increase in NIPPV use for children hospitalized with bronchiolitis.

### Limitations

This study has several limitations. As a study relying on administrative data, there is a risk of misclassification for certain variables, including outcomes. There are also limitations in which outcomes (eg, emergent ICU transfers) can be examined. Because information on patient-level receipt of HFNC is not reliably available in the PHIS database, we measured HFNC at the hospital level. We thus focused on comparisons by hospital-level adoption of HFNC protocols. The analysis mainly included children’s hospitals, making generalizability to other settings unclear. We adjusted for patient demographic variables available in the PHIS database, but other factors (eg, severity of illness) are incompletely captured in the database. Given the limitations of this study, future studies that examine which patients benefit from HFNC, which flow rates are most effective, and which patients require escalation of care beyond HFNC (including NIPPV support) are needed.

## Conclusions

In this cohort study of hospitalized children with bronchiolitis, transition to weight-based non-ICU HFNC protocols was associated with reduced ICU admission rates. There were no significant changes in mean hospital LOS or IMV use associated with the transition.

## Notes

Supplement.
Data Sharing Statement
