# Supplementary material for: Transition to Weight-Based High-Flow Nasal Cannula Use Outside of the ICU for Bronchiolitis
Source: JAMA Netw Open. 2024 Mar 18;7(3):e242722. doi: 10.1001/jamanetworkopen.2024.2722 (PMC10949097; doi:10.1001/jamanetworkopen.2024.2722)
Supplement: Supplement. — Data Sharing Statement [file jamanetwopen-e242722-s001.pdf]

Data Sharing Statement

Willer. Transition to Weight-Based High-Flow Nasal Cannula Use Outside of the ICU for Bronchiolitis. *JAMA Netw Open*. Published March 18, 2024.
doi:10.1001/jamanetworkopen.2024.2722

Data
Data available: No
